# Advent of oral medications for the treatment of hereditary angioedema

**DOI:** 10.1002/clt2.12391

**Published:** 2024-09-27

**Authors:** Anna Valerieva, Teresa Caballero, Markus Magerl, Joao P. Frade, Paul K. Audhya, Timothy Craig

**Affiliations:** ^1^ Department of Allergology Medical University of Sofia University Hospital “Alexandrovska” Sofia Bulgaria; ^2^ Servicio de Alergia Hospital Universitario la Paz IdiPAZ CIBERER U754 Madrid Spain; ^3^ Institute of Allergology IFA Charité – Universitätsmedizin Berlin Berlin Germany; ^4^ Fraunhofer Institute for Translational Medicine and Pharmacology ITMP Allergology and Immunology Berlin Germany; ^5^ KalVista Pharmaceuticals Cambridge Massachusetts USA; ^6^ Departments of Medicine, Pediatrics, and Biomedical Sciences Penn State University Hershey Pennsylvania USA; ^7^ Vinmec Medical Center Times City Hanoi Vietnam

**Keywords:** berotralstat, deucrictibant, hereditary angioedema, oral, sebetralstat

## Abstract

**Background:**

Hereditary angioedema (HAE) is a rare genetic disorder characterized by unpredictable, debilitating episodes of submucosal and/or subcutaneous tissue swelling, which may be life‐threatening depending on anatomic location. The two primary management strategies for HAE are ready access to effective on‐demand treatment in all patients and the prevention of attacks (short‐term prophylaxis [STP] and long‐term prophylaxis [LTP]) in appropriate patients. All approved on‐demand and most LTP medications require subcutaneous or intravenous administration. Injection‐related challenges include trypanophobia (fear of needles), difficulty with self‐administration, injection‐site reactions (e.g., pain, erythema, bleeding, bruising), and anxiety—all contributing to poor compliance and administration delays. Oral HAE treatments may improve outcomes by reducing treatment barriers.

**Aim:**

To review oral therapies, approved or in development, for on‐demand treatment and/or prevention of HAE attacks.

**Materials and Methods:**

To provide a comprehensive review, data was obtained from publicly available resources through a targeted PubMed literature review and supplemented by information provided on company websites (search cutoff of May 31, 2024).

**Results:**

Berotralstat, an oral plasma kallikrein (PKa) inhibitor, is approved for LTP. Sebetralstat, another PKa inhibitor, is the investigational first oral on‐demand HAE treatment to complete a phase 3 trial. Deucrictibant, an oral bradykinin B2 receptor antagonist, has completed phase 2 trials for on‐demand therapy and LTP. Several other oral PKa inhibitors (ATN249, VE‐4666, and VE‐4062) are in early development for LTP.

**Conclusion:**

Substantial advances have been made in the development of oral treatments for HAE. These treatments have the potential to improve and optimize clinical outcomes, satisfaction, and quality of life among patients with HAE.

## 1 INTRODUCTION

Hereditary angioedema (HAE) is a rare genetic disorder most commonly caused by either deficiency or dysfunction of C1 inhibitor (C1INH; HAE‐C1INH‐Type1 and HAE‐C1INH‐Type2, respectively). Less commonly, HAE with normal C1INH levels (HAE‐nC1INH) may occur. All types of HAE are characterized by unpredictable and recurrent debilitating attacks of subcutaneous (SC) and mucosal tissue swelling, which may be life‐threatening depending on the affected location.^1^, ^2^, ^3^ This article will focus on patients with HAE‐C1INH.

The goals of HAE treatment are to achieve total control of the disease and to normalize patients' lives.^4^ The World Allergy Organization (WAO)/European Academy of Allergy and Clinical Immunology (EAACI) guidelines for HAE management include the following recommendations: (1) all angioedema attacks should be considered for on‐demand treatment and should be treated as early as possible; (2) short‐term prophylaxis (STP) should be considered before medical, surgical, or dental procedures, as well as exposure to other events that could induce angioedema attacks; and (3) patients should be routinely evaluated for long‐term prophylaxis (LTP), considering disease activity, burden, control, and patient preference.

**FIGURE 1:**
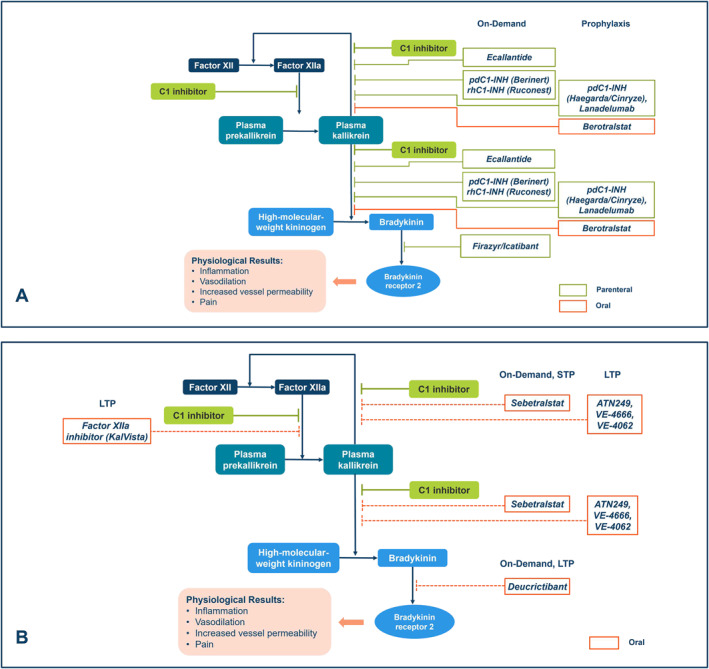
Activation and inhibition of the kallikrein‐kinin system by (A) approved agents^a^ and (B) investigational oral agents for HAE. ^a^Approval may vary according to geographic region/country. FXIIa, activated factor XII; LTP, long‐term prophylaxis; pdC1INH, plasma‐derived C1 inhibitor; rhC1INH, recombinant human C1 inhibitor; STP, short‐term prophylaxis.

Currently, nearly all approved HAE treatments require SC or intravenous (IV) administration (Figure 1A); however, the use of parenteral treatments for HAE has been associated with administration‐related anxiety (e.g., trypanophobia [fear of needles]), challenges related to administration (e.g., long preparation time, inconvenient storage/preparation),^5^, ^6^, ^7^ and adverse events that often accompany parenteral treatments, including injection‐site reactions.^7^, ^8^ Limitations of parenteral treatments contribute to poor compliance, including administration delays, and result in a high treatment burden and impaired quality of life (QoL) for many patients with HAE.^5^, ^6^, ^7^

No oral medication has been approved by regulatory agencies (United States [US] Food and Drug Administration [FDA] or European Medicines Agency [EMA]) for on‐demand treatment^9^; however, phase 2 and 3 clinical trials have been completed for oral agents (Table 1; Figure 1B).^10^, ^11^ As for STP, attenuated androgens (e.g., danazol and stanozolol) have been used in adults but are not FDA‐ or EMA‐approved and are considered second‐line alternatives to IV treatment with plasma‐derived C1 inhibitor (pdC1INH). Oral tranexamic acid, also not FDA‐ or EMA‐approved, has been used in the past for STP as well as LTP but is not recommended by guidelines given safety concerns and limited data suggesting benefit for LTP and proven minimal effectiveness for use as an on‐demand therapy.^4^, ^9^, ^12^, ^13^, ^14^ For LTP, the only oral agents approved by the FDA or EMA are berotralstat^15^, ^16^ and attenuated androgens (considered a second‐line treatment in the WAO/EAACI guidelines).^4^, ^12^

**TABLE 1:** Oral agents approved or in development for HAE.^a^

Generic name (investigational compound)	Mechanism of action	Manufacturer	Development status	Indication
Approved oral agents				
Prophylaxis				
Berotralstat (ORLADEYO)	PKa inhibitor	BioCryst pharmaceuticals	Phase 3 APeX‐2 trial completed (first regulatory approval 2020); phase 3 pediatric APeX‐P trial underway in patients aged 2 to <12 years (NCT05453968)	LTP in adults and pediatric patients aged ≥12 years
Attenuated androgens (Danazol)	Induction of aminopeptidase P activity and ↑C1INH synthesis and mRNA expression	Lannett company, Inc.	FDA‐approved for LTP	LTP (second‐line because of adverse events; adults only)
			Not EMA‐approved	
Investigational oral agents				
Prophylaxis				
Sebetralstat (KVD900)	PKa inhibitor	KalVista pharmaceuticals	KONFIDENT‐S (open‐label extension, includes STP)	STP in medical and dental procedures
Deucrictibant (PHVS719) extended‐release	B2R antagonist	Pharvaris	Phase 1 CHAPTER‐1 trial met its primary endpoint; open‐label portion is ongoing	LTP
ATN249	PKa inhibitor	Attune pharmaceuticals	Phase 1 clinical trial showed positive results in 2019; phase 2 trial planned but not yet initiated	LTP
Tranexamic acid (Lysteda)	Antifibrinolytic	Amring pharmaceuticals	Not FDA‐ or EMA‐approved	Not recommended for LTP; empirical use when nonandrogen options are not available or attenuated androgens are contraindicated
VE‐4666	PKa inhibitor	Verseon	Preclinical	LTP
VE‐4062	PKa inhibitor	Verseon	Preclinical	LTP
ATNXXX	PKa inhibitor	Attune pharmaceuticals	Early stages of discovery^b^	LTP
Avoralstat (formerly BCX4161)	PKa inhibitor	BioCryst pharmaceuticals	Results from phase 3 OPuS‐2 clinical trial published in 2018; efficacy not established, so BioCryst is no longer pursuing avoralstat for HAE	Not applicable
KVD824	PKa inhibitor	KalVista pharmaceuticals	KOMPLETE trial terminated because ↑LFTs seen in all treatment groups	Not applicable
On‐demand				
Sebetralstat (KVD900)	PKa inhibitor	KalVista pharmaceuticals	Phase 3 KONFIDENT trial completed	On‐demand treatment of HAE attacks
Deucrictibant (PHVS416) immediate‐release	B2R antagonist	Pharvaris	Phase 2 RAPIDe‐1 trial completed	On‐demand treatment of HAE attacks

Abbreviations: B2R, bradykinin B2 receptor; C1INH, C1 inhibitor; EMA, European medicines agency; FDA, United States food and drug administration; FXIIa, factor XIIa; HAE, hereditary angioedema; LTP, long‐term prophylaxis; PKa, plasma kallikrein; STP, short‐term prophylaxis.

^a^ Approval may vary according to geographic region/country.

^b^ Has not yet entered into preclinical studies.

Oral treatments for HAE could potentially optimize patient outcomes by reducing treatment barriers, improving patient adherence, and decreasing out‐of‐pocket costs for patients and their caregivers.^17^ Furthermore, some studies have revealed that patients with HAE would prefer oral medications over parenteral medications and that ease of access and fit with lifestyle are important factors to consider when selecting treatment.^18^, ^19^, ^20^ Oral on‐demand medications therefore represent an unmet need.^18^ To provide the most comprehensive understanding of the potential benefits of oral treatments, we have summarized data from all publicly available resources through a targeted literature review using PubMed and supplemented by information noted on company websites (search cutoff of May 31, 2024) to provide the most up‐to‐date information for each compound.

## 2 ORAL TREATMENTS

### 2.1 Mechanisms of action

Currently approved and investigational oral treatments for HAE target components of the kallikrein‐kinin system, including plasma kallikrein (PKa), activated factor XII (FXIIa), or bradykinin B2 receptor (B2R; Figure 1).^21^, ^22^ PKa inhibitors (e.g., berotralstat, sebetralstat, ATN249, VE‐4666, and VE‐4062) and FXIIa inhibitors prevent bradykinin generation by preventing cleavage of high molecular weight kininogen, whereas B2R antagonists (e.g., deucrictibant) block bradykinin binding.^21^

### 2.2 On‐demand treatment

Although no oral medication has been approved by the FDA or EMA for on‐demand treatment, one investigational medication has completed a phase 3 trial, and one investigational medication has completed a phase 2 trial (Table 1).

Sebetralstat (KVD900) is an oral PKa inhibitor in development by KalVista Pharmaceuticals. KONFIDENT (NCT05259917), a multinational, randomized, double‐blind, placebo‐controlled, 3‐way crossover, phase 3 trial was completed in December 2023. KONFIDENT enrolled 136 participants and evaluated the efficacy and safety of sebetralstat (300 and 600 mg) as an on‐demand therapy in adult and adolescent patients with HAE (including those with nonsevere laryngeal attacks). Participants were randomized in a 1:1:1:1:1:1 ratio to administer sebetralstat 300, 600 mg, or placebo for each eligible attack; a second dose could be administered at least 3 h after the first administration. Time to the beginning of symptom relief (defined as a Patient Global Impression of Change [PGI‐C] rating of “A Little Better” at two or more consecutive time points within 12 h of the first study drug administration) was the primary efficacy endpoint. Key secondary endpoints included a reduction in attack severity (defined as an improvement in Patient Global Impression of Severity [PGI‐S] rating [ratings range from “None” to “Very Severe”] at two or more consecutive time points within 12 h of the first study drug administration) and complete resolution of attack (defined as a rating of “None” on the PGI‐S scale within 24 h of the first study drug administration).^10^

**FIGURE 2:**
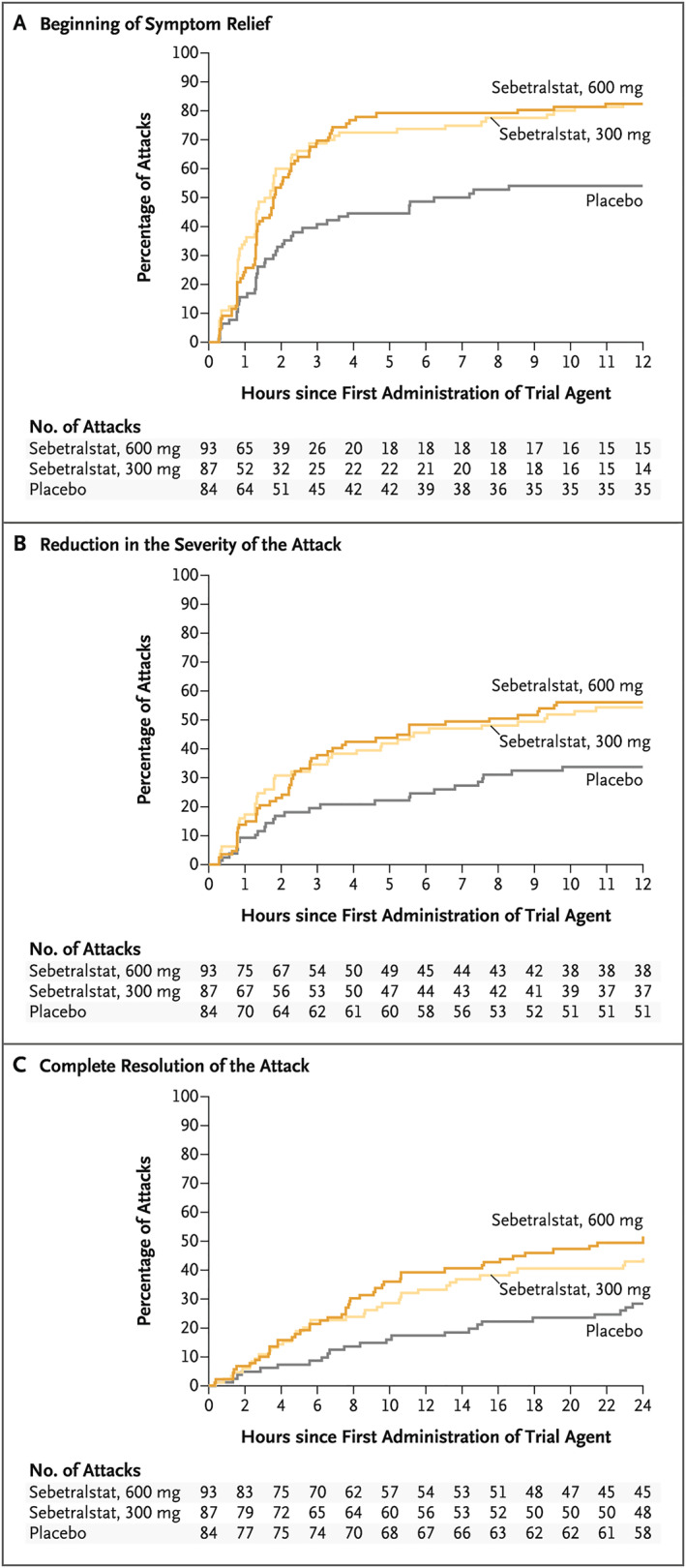
Primary and key secondary endpoints from a phase 3 study for sebetralstat. (A) Beginning of symptom relief (primary endpoint; defined as a rating of “A Little Better” on the seven‐point PGI‐C at ≥2 consecutive time points within 12 h of first study drug administration) as assessed in a time‐to‐event analysis. (B) Reduction in the severity of the attack (defined as an improved rating on the PGI‐S scale at ≥2 consecutive time points within 12 h of first study drug administration) as assessed in a time‐to‐event analysis. (C) Complete resolution of the attack (defined as a rating of “None” on the PGI‐S scale within 24 h of the first administration) as assessed in a time‐to‐event analysis. PGI‐C, Patient Global Impression of Change; PGI‐S, Patient Global Impression of Severity; Reprinted from *New England Journal of Medicine*, Riedl MA, Farkas H, Aygören‐Pürsün E, et al. Oral sebetralstat for on‐demand treatment of hereditary angioedema attacks, 10.1056/NEJMoa2314192, Copyright (2024), with permission from Elsevier.^10^

In the KONFIDENT trial, 110 patients treated 264 attacks with sebetralstat 300 mg (*n* = 87), 600 mg (*n* = 93), and placebo (*n* = 84). The time to beginning of symptom relief was significantly shorter with sebetralstat versus placebo (*p* < 0.001 for 300 mg; *p* = 0.001 for 600 mg; Figure 2A). Median time to beginning of symptom relief was 1.61 h (interquartile range [IQR], 0.78–7.04) for 300 mg, 1.79 h (IQR, 1.02–3.79) for 600 mg, and 6.72 h (IQR, 1.34‐>12) for placebo. Results for the key secondary endpoints are shown in Figures 2B and C. There were no reports of serious adverse events (SAEs) related to treatment or treatment discontinuations due to adverse events.^10^ Results from KONFIDENT were consistent with those from the phase 2 trial for sebetralstat.^23^ The long‐term safety and efficacy of sebetralstat are being studied in the KONFIDENT‐S trial (NCT05505916), an ongoing, 2‐year, open‐label extension of KONFIDENT.^24^ Phase 1 results for an orally disintegrating tablet formulation for sebetralstat have also been presented.^25^

Deucrictibant (PHVS416), an oral B2R antagonist developed by Pharvaris, was recently studied in a phase 2 trial.^11^, ^26^ RAPIDe‐1 (NCT04618211) was a multinational, randomized, double‐blind, placebo‐controlled, crossover, dose‐ranging, phase 2 trial.^11^, ^26^ The primary endpoint was the change in the mean 3‐symptom composite (skin pain, skin swelling, and abdominal pain) visual analog scale (VAS)‐3 score during HAE attacks at 4 h. Secondary endpoints included time to onset of symptom relief, time to ≥50% reduction in VAS‐3, and time to “almost complete” or complete resolution of symptoms. Additional endpoints included change in mean symptom severity score (MSCS) at 4 h, treatment outcome score (TOS) at 4 h, and use of rescue medication. One hundred forty‐seven HAE attacks were treated with PHVS416 10 mg (*n* = 37), 20 mg (*n* = 28), 30 mg (*n* = 31), or placebo (*n* = 51) in 62 patients.^27^

**FIGURE 3:**
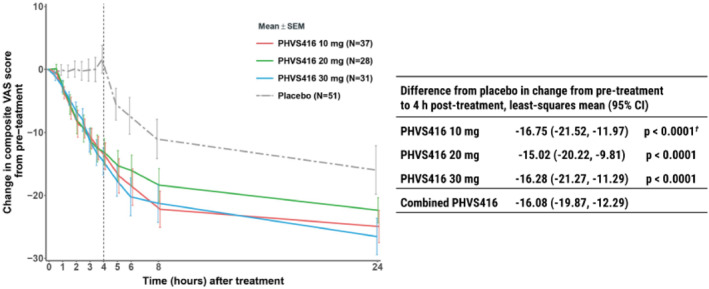
Primary endpoint results (reduction of attack symptoms by VAS‐3) for the RAPIDe‐1 phase 2 trial of deucrictibant. Median VAS‐3 at baseline ranged from 24.33 to 27.00 across deucrictibant IR capsule doses (10, 20, and 30 mg). The figure is based on the descriptive summary of the mean and SEM. Least square means differences, CIs, and *p*‐values come from an MMRM. Data after rescue medication use are not included. The combined PHVS416 result is based on post hoc analysis using a similar MMRM with all 3 active doses combined versus placebo. ^†^Nominal *p*‐value; VAS assessed every 30 min up to 4 h posttreatment, then at 5, 6, 8, 24, and 48 h. CI, confidence interval; IR, immediate release; MMRM, mixed‐effects model with repeated measures; N, the number of attacks in the mITT Analysis Set; SEM, standard error of the mean; VAS‐3, electronically captured, numerically assisted visual analog scale. Reproduced from Maurer et al, 2023.^11^, ^28^

At 4 h, PHVS416 significantly reduced symptoms of HAE attacks based on changes in VAS‐3 compared with placebo (10 mg: −16.75; 20 mg: −15.02; 30 mg: −16.28; *p* < 0.0001 for all 3 doses; Figure 3). Results for key secondary endpoints are summarized in Table 2.^11^, ^28^ PHVS416 was generally well tolerated.^27^

**TABLE 2:** Results of key secondary efficacy endpoints for the RAPIDe‐1 phase 2 trial for deucrictibant.

Endpoint	Placebo *n* = 51	PHVS416 10 mg *n* = 37	PHVS416 20 mg *n* = 28	PHVS416 30 mg *n* = 31	Combined PHVS416^a^ *n* = 96
Time to onset of symptom relief by VAS‐3 ≥30% reduction^b^					
Median time in hours (95% CI)	8.0 (7.6, 46.9)	2.1 (1.5, 2.9)	2.7 (1.9, 3.5)	2.5 (1.9, 3.8)	2.4 (2.0, 2.9)
Hazard ratio		3.81	3.08	3.61	
*p* value		<0.0001	0.0021	<0.0001	
Time to VAS‐3 ≥50% reduction^b^					
Median time in hours (95% CI)	22.8 (20.0, 24.1)	3.3 (2.4, 3.9)	4.0 (2.9, 6.0)	4.0 (3.3, 5.8)	3.9 (3.0, 4.8)
Hazard ratio		4.55	3.65	3.87	
*p* value		<0.0001	0.0003	<0.0001	
Time to almost complete or complete symptom relief by VAS‐3^b^					
Median time in hours (95% CI)	42.0 (22.0, 48.1)	5.8 (3.6, 7.5)	20.0 (4.5, 20.0)	20.0 (6.0, 20.1)	7.5 (5.9, 20.0)
Hazard ratio		5.09	2.25	2.65	
*p* value		<0.0001	0.0127	0.0001	
Change in MSCS^c^ score at 4 hours^d^					
Least‐squares mean difference: PHVS416‐placebo		−0.79	−0.61	−0.39	−0.61
*p* value		<0.0001	0.0008	0.0291	
TOS^e^ at 4 hours^e^					
Least‐squares mean difference: PHVS416‐placebo		64.13	62.69	71.06	66.05
*p* value		<0.0001	<0.0001	<0.0001	

*Note*: *p‐*values for PHVS416 20 and 30 mg are based on statistical tests in the prespecified multiple comparison procedure; other *p* values are nominal.

Abbreviations: CI, confidence interval; MSCS, mean symptom severity score; *n*, number of attacks included in the modified intent to treat analysis set; TOS, treatment outcome score; VAS, visual analog scale.

^a^ The combined PHVS416 *results* are based on post hoc analyses to provide a reference of the result by pooling all 3 active doses.

^b^ Hazard ratios and *p*‐values are based on marginal Cox proportional hazard models.

^c^ Minimal clinically important difference for MSCS = −0.30.

^d^ *p* values are based on mixed‐effects models for repeated measures.

^e^ Minimal clinically important difference for TOS = 30.

*Source:* Reproduced from Maurer et al, 2023.^11^, ^28^

During phase 2, the FDA put the program on clinical hold, but this was lifted, and Pharvaris initiated a phase 3 trial (RAPIDe‐3; NCT06343779). The primary endpoint will be the time to onset of symptom relief, as measured by the PGI‐C, and will include patients with nonsevere laryngeal attacks.^29^

The efficacy and safety of berotralstat, an oral PKa inhibitor developed by BioCryst Pharmaceuticals, was evaluated as an on‐demand treatment in a randomized, double‐blind, placebo‐controlled, dose‐ranging, phase 2 trial (ZENITH‐1). Only results for the completed 750 mg dose cohort have been published. Thirty‐three adult patients with HAE were treated with berotralstat for 95 attacks. At 4 h postdose, the percentage of HAE attacks that were stable or improved based on a composite VAS was 67.7% and 46.7% for berotralstat versus placebo (*p* = 0.0387; Table 3). At 24 h postdose, the percentage of patients with no or mild symptoms based on a patient global assessment was 64.1% and 32.3% for berotralstat versus placebo (*p* = 0.0038). Rescue medication was used in 61.3% of placebo‐treated patients versus 29.7% of berotralstat‐treated patients (*p* = 0.0029). Berotralstat was well tolerated (as defined by no grade 3/4 adverse events or laboratory abnormalities reported).^30^ Although results from the ZENITH‐1 study may have supported further evaluation of the 750 mg dose level, dosages higher than 150 mg are not recommended due to a concentration‐dependent increase in QT prolongation.^15^

**TABLE 3:** Efficacy results for part 1 (750 mg) of the phase 2 ZENITH‐1 trial for berotralstat.

Endpoint	Berotralstat 750 mg *n* = 64 attacks	Placebo *n* = 31 attacks	Difference	*p* value
Least square means change from baseline in VAS score through 4 hours^a^	−3.9	+3.1	−6.98	0.0024
Proportion of attacks requiring standard‐of‐care treatment through 24 h	29.7%	61.3%	−31.6%	0.0029
Proportion of attacks with no or mild symptoms through 24 hours^a^	64.1%	32.3%	+31.8%	0.0038
Time to standard‐of‐care acute attack treatment (median)	>24 h	14 h	>+10 h	0.0043
Proportion of attacks with improved or stable symptoms through 24 hours^a^	64.1%	35.5%	+28.6%	0.0092
Proportion of attacks with improved or stable VAS score through 24 hours^a^	62.5%	35.5%	+27.0%	0.0125
Proportion of attacks with improved or stable symptoms through 4 hours^a^	82.3%	60.0%	+22.3%	0.0192
Proportion of attacks with improved or stable VAS score through 4 hours^a^	67.7%	46.7%	+21.0%	0.0387
Time to stable or improved VAS (median)^a^	1 h	2 h	−1 h	0.0452
Proportion of attacks with no or mild symptoms through 4 hours^a^	69.4%	50.0%	+19.4%	0.0552
Time to ≥50% reduction in VAS through 24 h (median)^a^	8 h	24 h	−16 h	0.0671
Time to initial symptom relief (median)^a^	5.1 h	19.4 h	−14.3 h	0.0978
Time to almost complete symptom relief (median)^a^	23.1 h	23.6 h	−0.5 h	0.6767
Time to complete symptom relief (median)^a^	35.1 h	41.3 h	−6.2 h	0.8900

Abbreviation: VAS, visual analog scale.

^a^ Data censored for least square means or endpoint = failure (proportions) for time points after a subject took rescue medication.

*Source:* Reproduced from Longhurst et al, 2019.^30^

### 2.3 Short‐term prophylaxis

Attenuated androgens (e.g., danazol and stanozolol) have been used in adults for STP but are not FDA‐ or EMA‐approved for this purpose; however, they are considered alternatives to IV pdC1INH if unavailable.^4^, ^9^, ^12^ In a study of 12 patients with HAE undergoing maxillofacial or dental procedures, STP with danazol 600 mg/day for 4 days presurgery and 4 days postsurgery effectively prevented HAE attacks in all 12 patients, and no adverse events were reported.^31^ In a long‐term observational study evaluating oral danazol (2.5–10 mg/kg/day for 5 days before and 2 days after procedure; *n* = 38), oral tranexamic acid (20–40 mg/kg/day for 5 days before and 2 days after procedure; *n* = 9), and IV pdC1INH (500 IU 1 h before procedure; *n* = 87) in patients with HAE who were undergoing dental or medical diagnostic/surgical interventions, 13%, 33%, and 6% with danazol, tranexamic acid, and pdC1INH experienced an HAE attack postprocedure despite STP (*p* = 0.0096 for pdC1INH vs. the oral agents).^32^ In another observational study evaluating oral tranexamic acid for STP (1 g every 6 h for 2 days before and 2 days after the procedure) in patients with HAE who were undergoing oropharyngeal or general surgical procedures, none of the 14 patients experienced an HAE attack postprocedure.^13^

Short‐term courses of attenuated androgens usually have limited side effects; however, frequent use may have adverse effects approaching those of LTP.^4^, ^9^ When attenuated androgens are used for STP, they generally are started 5–7 days before the stressor/procedure and continued for 2–3 days afterward.^4^, ^9^, ^33^

Sebetralstat is also being studied for STP in the open‐label KONFIDENT‐S trial in patients aged ≥12 years with HAE‐C1INH‐Type1 or HAE‐C1INH‐Type2. KONFIDENT‐S is the first prospective trial to evaluate an oral therapy for STP. To assess the safety and efficacy of sebetralstat as STP, patients will administer the first of the 3‐dose course of 600 mg sebetralstat approximately 1 h before a surgical, medical, or dental procedure and then 2 doses approximately every 6 h thereafter.^2^, ^34^

### 2.4 Long‐term prophylaxis

For LTP, oral medications have been approved by the FDA, and several others are in various stages of clinical development.

#### 2.4.1 Approved treatments

**FIGURE 4:**
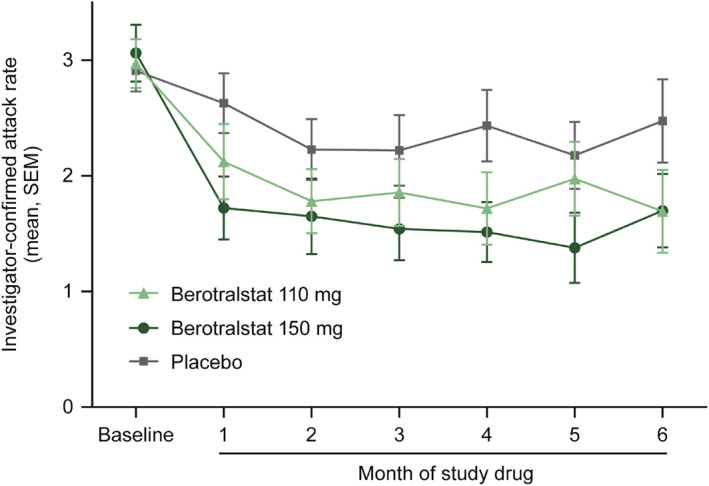
Mean investigator‐confirmed attack rate by months in the intent‐to‐treat population from the APeX‐2 trial for berotralstat. SEM, standard error of the mean. Reprinted from *J Allergy Clin Immunol*, 148(1), Zuraw B, Lumry WR, Johnston DT, et al., Oral once‐daily berotralstat for the prevention of hereditary angioedema attacks: a randomized, double‐blind, placebo‐controlled phase 3 trial, 164–172, Copyright 2021, with permission from Elsevier.^37^

Berotralstat is an oral PKa inhibitor that was developed by BioCryst and is the first oral treatment approved by various regulatory agencies (the FDA, EMA, UK Medicines and Healthcare products Regulatory Agency, and the Ministry of Health, Labor and Welfare in Japan) for LTP in adults and pediatric patients aged 12 years and older.^15^, ^16^, ^35^, ^36^ In the randomized, double‐blind, placebo‐controlled, parallel‐group, multinational, pivotal phase 3 APeX‐2 trial, patients with HAE were randomized 1:1:1 to receive berotralstat doses of 150 mg (*n* = 40) or 110 mg (*n* = 41) or placebo (*n* = 40) orally once daily. Berotralstat treatment resulted in significant reductions in HAE attack rates versus placebo after 24 weeks of treatment (primary efficacy endpoint; 110 mg: 1.65 attacks/month [*p* = 0.024]; 150 mg: 1.31 attacks/month [*p* < 0.001]; placebo: 2.35 attacks/month; Table 4). The reduction in the mean attack rate began within the first month and was sustained throughout the 24‐week period (Figure 4). The change from baseline in angioedema‐QoL (AE‐QoL) total scores at week 24 was not significant in either dose group versus placebo, and formal statistical analysis was not performed on other secondary endpoints. Both doses were generally well tolerated (most common treatment‐emergent adverse events [TEAEs; ≥10% in any treatment group] were abdominal pain, vomiting, diarrhea, and back pain). Overall, 5 patients discontinued treatment because of TEAEs (3 in the 110 mg group, 1 each in the other 2 groups), all of which were considered possibly or probably related to the study drug except for 1 (110 mg group). Only 1 grade 3/4 laboratory abnormality occurred (asymptomatic grade 4 elevation in alanine aminotransferase level in the 150 mg group).^37^

**TABLE 4:** Summary of endpoints and additional analyses, intent‐to‐treat population from the APeX‐2 trial for berotralstat.

Endpoint	Berotralstat 110 mg *n* = 41	Berotralstat 150 mg *n* = 40	Placebo *n* = 40
Primary			
Estimated monthly investigator‐confirmed attack rate through week 24^a^	1.65	1.31	2.35
Attack rate ratio relative to placebo (95% CI)	0.70 (0.51–0.95)	0.56 (0.41–0.77)	–
*p* value	0.24	<0.001	–
Secondary			
Change from baseline to week 24 in AE‐QoL total score, least square means (standard error)^b^	−12.46 (2.53)	−14.59 (2.59)	−9.69 (2.64)
Difference from placebo, least square means (95% CI)	−2.77 (−10.08 to 4.53)	−4.90 (−12.23 to 2.43)	–
*p* value	0.453	0.188	–
Proportion of days with angioedema symptoms, least square means (standard error)^c^	0.134 (0.019)	0.119 (0.019)	0.197 (0.020)
Difference from placebo, least square means (95% CI)	−0.062 (−0.117 to −0.008)	−0.078 (0.133 to −0.023)	–
Nominal *p* value	0.025	0.006	–
Estimated monthly confirmed attack rate over the effective dosing period (day 8 to week 24)^a^ ^,^ ^d^	1.65	1.27	2.38
Attack rate ratio relative to placebo (95% CI)	0.70 (0.51–0.96)	0.54 (0.39–0.74)	–
Nominal *p* value	0.26	<0.001	–

Abbreviations: AE‐QoL, angioedema‐quality of life; CI, confidence interval.

^a^ Investigator‐confirmed attack rate is defined as the total number of investigator‐confirmed HAE attacks experienced in the entire Part 1 dosing period. Statistical analysis is based on a negative binomial regression model in which the number of investigator‐confirmed attacks is included as the dependent variable, the treatment is included as a fixed effect, the baseline investigator‐confirmed attack rate is included as a covariate, and the logarithm of the duration of treatment is included as an offset variable.

^b^ The AE‐QoL scores range from 0 (best) to 100 (worst). Statistical analysis is based on a mixed‐model repeated measures analysis with baseline investigator‐confirmed attack rate, baseline AE‐QoL, treatment, visit, and visit‐by‐treatment interaction included as fixed effects. The patient is included as a random effect.

^c^ The proportion of days with angioedema symptoms due to investigator‐confirmed attacks is based on the number of days with reported symptoms from investigator‐confirmed attacks in Part 1 and the number of days the patient received treatment in Part 1. Statistical analysis is based on an analysis of the covariance model with baseline investigator‐confirmed attack rate as a covariate and treatment included as a fixed effect.

^d^ The effective dosing period is the steady‐state dosing period defined as days 8–168.

*Source:* Reprinted from *J Allergy Clin Immunol*, 148(1), Zuraw B, Lumry WR, Johnston DT, et al., Oral once‐daily berotralstat for the prevention of hereditary angioedema attacks: a randomized, double‐blind, placebo‐controlled phase 3 trial, 164–172, Copyright 2021, with permission from Elsevier.^37^

In part 2 of APEX‐2, patients randomized to placebo in part 1 were rerandomized 1:1 to berotralstat 150 mg (*n* = 17) or berotralstat 110 mg (*n* = 17), and those who received active treatment in part 1 continued at the same dose through week 48 (*n* = 37 for both dose groups). The primary endpoint was the safety and tolerability of berotralstat; secondary endpoints included effectiveness, QoL, and patient satisfaction (based on the Treatment Satisfaction Questionnaire for Medicine [TSQM]) with berotralstat from week 24 to week 48. Berotralstat was generally well tolerated (most common TEAEs [≥10% during parts 1 and 2 in any treatment group] were upper respiratory tract infection, nausea, abdominal pain, dyspepsia, diarrhea, vomiting, headache, flatulence, gastroesophageal reflux disease, and back pain). Five drug‐related grade 3 TEAEs were reported. Three patients discontinued treatment in part 2 because of TEAEs (150 mg group: 2 due to anal incontinence; 110 mg group: 1 due to nausea). There were no drug‐related grade 4 TEAEs or SAEs.^38^

In APEX‐2 part 3, patients received open‐label treatment with berotralstat 150 mg for up to an additional 240 weeks (*n* = 81). The primary endpoint was long‐term safety and tolerability from week 48. Secondary endpoints included HAE attack rates, QoL, and patient satisfaction. The most common TEAEs (≥5%) were nasopharyngitis, urinary tract infection, abdominal pain, arthralgia, coronavirus infection, and diarrhea. One patient experienced a drug‐related grade 3 adverse event (abdominal pain). There were no drug‐related grade 3/4 laboratory abnormalities or SAEs.^39^

In the randomized, double‐blind, placebo‐controlled, parallel‐group, phase 3 APeX‐J trial, 19 patients were randomized to receive once‐daily oral berotralstat 110 mg (*n* = 6), 150 mg (*n* = 7), or placebo (*n* = 6) for 24 weeks.^40^ The primary endpoint was the rate of expert‐confirmed HAE attacks over the 24‐week treatment period. Secondary endpoints included the number and proportion of days with HAE symptoms, the rate of expert‐confirmed HAE attacks during the effective treatment period, and the change from baseline in AE‐QoL scores at week 24. Expert‐confirmed attacks decreased by 49% in the 150 mg group (*p* = 0.003 vs. placebo) and by 25% in the 110 mg group (*p* = 0.181 vs. placebo). Drug‐related TEAEs were reported in 2 patients in each treatment group (150 mg: abdominal pain upper, gastritis, pyrexia, somnolence; 110 mg: abdominal discomfort, diarrhea, nausea, headache). No grade 3/4 TEAEs were reported.^40^

Overall, in the APeX‐2 and APeX‐J trials, the benefit‐to‐risk profile was better for the 150 mg dose^37^, ^40^; therefore, the recommended dose of berotralstat is one 150 mg capsule taken once daily.^15^, ^16^, ^35^ Another trial, the open‐label, nonrandomized, multinational APeX‐S trial, was designed to evaluate the long‐term safety and efficacy of berotralstat (150 mg [*n* = 127] and 110 mg [*n* = 100]) as LTP for HAE through 48 weeks of treatment. Results from a planned interim analysis showed that both doses were well tolerated (most common drug‐related adverse events were abdominal pain, diarrhea, and nausea), and the mean rates of HAE attacks were low throughout the analysis period (month 1: 1.2 attacks/month for 150 mg, 1.1 attacks/month for 110 mg; month 12: 0.8 attacks/month for both groups).^41^ Subsequently, the APeX‐S was completed.^42^ Overall, 57 (25%) patients discontinued the study drug before 48 weeks, mostly due to perceived lack of efficacy (*n* = 28 [12%]) or adverse events (*n* = 19 [8%]). Furthermore, 67 (30%) patients had not yet reached 48 weeks of dosing (23 in the 150 mg group, 44 in the 110 mg group). Therefore, the efficacy and safety results of this trial are difficult to interpret.^41^

A separate analysis of the APeX‐S study was conducted in the US to evaluate treatment satisfaction in 34 patients with HAE who switched from prior injectable treatment (lanadelumab [*n* = 21], SC C1INH [*n* = 12], or IV C1INH [*n* = 1]) to berotralstat 150 mg once daily. Patient satisfaction was measured using the TSQM (higher scores on a scale of 0–100 indicated greater satisfaction). From baseline to month 12, improvements were observed in all TSQM domains, including global satisfaction (+16.7 points), convenience (+30.4 points), effectiveness (+1.1 points), and side effects (+6.4 points). More specifically, global satisfaction increased by 15.0 points in patients who switched from lanadelumab, 15.6 points in patients who switched from SC C1INH, and 64.3 points in patients who switched from IV C1INH. The statistical significance of these changes was not assessed.^43^

Based on the WAO/EAACI guidelines for the management of HAE, berotralstat is the only oral agent recommended as first‐line therapy (81% agreement). The following should be considered for berotralstat use: dose reductions from 150 to 110 mg may be required in patients with hepatic impairment, concomitant use of P‐glycoprotein or breast cancer‐resistant glycoprotein inhibitors, or when patients experience gastrointestinal adverse events.^4^ The pharmacokinetic and safety profiles of berotralstat in pediatric patients aged 2 to <12 years are being studied in the phase 3 single‐arm open‐label APeX‐P trial (NCT05453968).^44^

Attenuated androgens are FDA‐approved for LTP in adult patients with HAE^9^, ^12^; however, they are considered second‐line treatment due to adverse events.^4^, ^9^, ^33^ In 3 separate trials, investigators evaluated the efficacy and safety of LTP with attenuated androgens in patients with HAE. In one of these trials, the efficacy of LTP with ≥1 attenuated androgens in 14 adult patients with HAE was evaluated. Twelve patients received danazol 600 mg/day intermittently for the first month (5 days on and 5 days off). If HAE attacks did not occur, the dose was tapered to the minimum effective dose for each patient. Six patients received stanozolol (4–12 mg/day [4 of these patients had been treated with danazol]), 3 had danazol replaced with quinbolone (40 mg/day), mesterolone (100 mg/day), or nandrolone decanoate (50 mg/week). Only danazol and stanozolol were effective in preventing HAE attacks.^45^ In another trial (*N* = 69), LTP with danazol (initial 600 mg/day dose reduced at 100–200 mg/day increments at 6‐ to 12‐week intervals to a level that consistently controlled symptoms) reduced the frequency and severity of attacks in all patients. However, prolonged use of danazol was associated with a high incidence of adverse effects, including weight gain (38%), menometrorrhagia (34%), myalgias/cramps (28%), headaches (22%), and alopecia (17%).^46^ In a small randomized, double‐blind, placebo‐controlled trial evaluating the use of danazol in 9 adults with HAE, danazol (200 mg 3 times daily) was significantly more effective than placebo in preventing HAE attacks (*p* < 0.001). Weight gain was reported in all 9 patients, and menstrual irregularities were reported in all women (*n* = 5) in the trial.^47^ It should be noted that the dosing of 200 mg 3 times a day exceeds the recommended dose of 200 mg/day by both the American and Global HAE guidelines, and this high dose is assumed to have contributed to the adverse effects.^4^, ^9^

Investigators also evaluated the effects of LTP with danazol (33–200 mg/day for 5–195 months) on lipid profiles and atherogenic indices in 37 patients with HAE compared with untreated patients with HAE (*n* = 27) and healthy participants (*n* = 66). Danazol‐treated patients had significantly higher low‐density lipoprotein (LDL) and apoprotein B‐100 (Apo B‐100) levels and significantly lower high‐density lipoprotein (HDL) and apoprotein A‐1 levels (Apo A1) compared with control groups (*p* ≤ 0.046). In addition, both atherogenic indices (ratios of LDL/HDL and Apo B‐100/Apo A‐1) were significantly higher in danazol‐treated patients compared with control groups (*p* ≤ 0.0013).^48^

#### 2.4.2 Treatments not‐approved for LTP use

Tranexamic acid, an antifibrinolytic, is not FDA‐ or EMA‐approved for HAE. However, guidelines recommend that tranexamic acid may be used when nonandrogen agents are unavailable, when androgens are contraindicated, or when the patient will only accept oral therapy.^4^, ^13^, ^14^ In a placebo‐controlled study of LTP with tranexamic acid (1 g, 3 times daily), seven of 18 patients had complete/almost complete cessation of attacks, 4 patients had a lower frequency and severity of attacks, and 1 patient had no response. The frequency of attacks was significantly lower with tranexamic acid versus placebo (*p* < 0.005). Minimal adverse events were reported (mild abdominal discomfort and diarrhea [*n* = 4]; pruritus ani [*n* = 1]).^14^

#### 2.4.3 In development

The CHAPTER‐1 trial (NCT05047185) was a phase 2 proof‐of‐concept trial in 34 patients with HAE designed to assess the safety and efficacy of 2 doses of immediate‐release deucrictibant (PHVS416 10 mg [low] and 20 mg [high]) twice daily as LTP for HAE over 12 weeks.^49^, ^50^ CHAPTER‐1 met its primary endpoint (monthly attack rate reduced by 84.5% vs. placebo [*p* = 0.0008] in the high‐dose group), and both doses of PHVS416 appeared to have been well tolerated. The open‐label portion of the trial at the 40 mg/day dose is ongoing.^51^ As the FDA recently removed the clinical hold on deucrictibant for LTP in January 2024, results from these trials will inform design of a phase 3 trial in which an extended‐release once daily formulation of deucrictibant (PHVS719) is expected to be evaluated as LTP for HAE.^49^, ^52^, ^53^ The extended‐release formulation was previously studied in an open‐label, crossover, single‐dose, phase 1 trial and demonstrated drug exposure starting within 3 h of administration and lasting for at least 28 h.^54^

Another oral PKa inhibitor, ATN249, is in clinical development by Attune Pharmaceuticals for LTP. A phase 1 trial showed that in 48 healthy participants, ATN249 exhibited dose‐dependent inhibition of PKa activity and a favorable safety profile; a phase 2 trial was in planning but has not been initiated.^55^, ^56^, ^57^, ^58^ Attune Pharmaceuticals has another oral PKa inhibitor, presently known as ATNXXX, in the early stages of discovery for LTP.^57^ Other oral agents in preclinical development for LTP include PKa inhibitors by Verseon International Corporation (VE‐4666 and VE‐4062).^59^

Avoralstat was another oral PKa inhibitor developed by Biocryst Pharmaceuticals that was studied for use as an LTP; however, efficacy was not demonstrated in the phase 3 OPuS‐2 trial, despite following the positive phase 2 OPuS‐1 trial. It is no longer being evaluated.^60^ KVD824 is another oral PKa inhibitor developed by KalVista Pharmaceuticals that was studied for use as an LTP; however, after safety concerns were raised in the phase 2 KOMPLETE trial, the trial was terminated and KVD824 is no longer being evaluated.^61^

In summary, several exciting new oral HAE treatments are on the horizon for on‐demand use, STP, and LTP, which have the potential to reduce the treatment burden and improve compliance and treatment satisfaction in patients with HAE. Initially, adults and adolescents will benefit from the new therapy options, but conceivably, soon after, younger children may benefit as well, as they can be expected to have a particularly substantial benefit from oral treatments.

It is important to note that there are several limitations to this article. First, this is not a systematic review. Second, publicly available information on many of the agents covered in this article is currently limited. Much of the clinical research on these agents has not yet been published in a peer‐reviewed format. Indeed, to be current, information on company websites was leveraged, which may be incomplete. Therefore, continued evaluation of the published literature as these agents progress through their respective development programs will be necessary to stay current on the advent of oral treatments in HAE.

## 3 CONCLUSION

Substantial advances have been made in the development of oral treatments for HAE, with international regulatory approvals of berotralstat for LTP and the first oral therapy, sebetralstat, completing phase 3 clinical development for on‐demand treatment. Additional agents are under investigation in preclinical, phase 1, and phase 2 programs. Oral treatments have the potential to improve and optimize clinical outcomes, satisfaction, and QoL among patients with HAE.

## AUTHOR CONTRIBUTIONS

**Anna Valerieva**: Conceptualization; investigation; writing—review & editing. **Teresa Caballero**: Conceptualization; validation; writing—review & editing. **Markus Magerl**: Conceptualization; methodology; writing—review & editing. **Joao P. Frade**: Investigation; validation; writing—review & editing. **Paul K. Audhya**: Conceptualization; methodology; validation; writing—review & editing; supervision. **Timothy Craig**: Conceptualization; methodology; validation; writing—review & editing; supervision.

## CONFLICT OF INTEREST STATEMENT

T.J.C. served as a speaker and researcher for Biomarin, CSL Behring, Regeneron, Kalvista and Takeda; researcher for Astria, Ionis, KalVista, Intellia, GSK and Pharvaris; speaker for Grifols; consultant for Astria, BioCryst, Biomarin, CSL Behring, Intellia, and KalVista; Director for ACARE International Hereditary Angioedema Center and Alpha‐1 Resource Center; member of the Medical Advisory Board for the HAE‐A. A.V. has received consultancy/speaker honoraria/meeting sponsorship from, or collaborated in research with, Pharming Group NV, Takeda/Shire, Sobi, CSL Behring, Pharvaris, and Ionis. T.C. has participated on advisory boards from Austria, BioCryst, CSL Behring, KalVista, Novartis, Pharming NV, Pharvaris, and Takeda and is a member of speakers’ bureaus for CSL Behring, Novartis, Pharming and Takeda. She has received grants or honoraria from BioCryst, CSL Behring, Kalvista, Novartis, Pharming NV, and Takeda and funding to attend conferences and educational events from BioCryst, CSL Behring, Novartis, Pharming, and Takeda. She is a clinical trial/registry/study investigator for BioCryst, Biomarin, CSL Behring, Ionis, KalVista, Novartis, Pharming NV, and Takeda and a researcher on the Instituto de Investigación Hospital Universitario La Paz (IdiPAZ) program for promoting research activities. M.M. received research grant support and/or speaker/consultancy fees from and/or is/was investigator in clinical studies for BioCryst, CSL Behring, Intellia, KalVista, Novartis, Octapharma, Pharming, Pharvaris, Takeda/Shire and Ionis. J.F. and P.A. are employees of KalVista Pharmaceuticals.

## Data Availability

Data sharing not applicable to this article as no datasets were generated or analyzed during the current study.
